# [Retracted] microRNA‑195 inhibits cell proliferation in bladder cancer via inhibition of cell division control protein 42 homolog/signal transducer and activator of transcription‑3 signaling

**DOI:** 10.3892/etm.2023.12366

**Published:** 2023-12-28

**Authors:** Cheng Zhao, Lin Qi, Minfeng Chen, Longfei Liu, Weiqian Yan, Shiyu Tong, Xiongbing Zu

Exp Ther Med 10:1103–1108, 2015; DOI: 10.3892/etm.2015.2633

Following the publication of the above paper, it was drawn to the Editors’ attention by a concerned reader that the western blotting data shown in Figs. 1B, 3 and 4B were strikingly similar to data appearing in different form in other articles written by different authors at different research institutes that had either already been published elsewhere prior to the submission of this paper to *Experimental and Therapeutic Medicine*, or were under consideration for publication at around the same time. Moreover, there appeared to be the duplication of a protein band within Figs. 1B and 4B, where the results from differently performed experiments were intended to have been shown. In view of the fact that certain of these data had already apparently been published previously, the Editor of *Experimental and Therapeutic Medicine* has decided that this paper should be retracted from the Journal. The authors were asked for an explanation to account for these concerns, but the Editorial Office did not receive a reply. The Editor apologizes to the readership for any inconvenience caused.

